# Saccade-related neural communication in the human medial temporal lobe is modulated by the social relevance of stimuli

**DOI:** 10.1126/sciadv.abl6037

**Published:** 2022-03-18

**Authors:** Tobias Staudigl, Juri Minxha, Adam N. Mamelak, Katalin M. Gothard, Ueli Rutishauser

**Affiliations:** 1 Department of Neurosurgery, Cedars-Sinai Medical Center, Los Angeles, CA 90048, USA.; 2 Department of Psychology, Ludwig-Maximilians-University, Munich, Germany.; 3 Division of Biology and Biological Engineering, California Institute of Technology, Pasadena, CA 91125, USA.; 4 Center for Theoretical Neuroscience, Columbia University, New York, NY 10027, USA.; 5 Department of Physiology, College of Medicine, University of Arizona, Tucscon, AZ 85724, USA.; 6 Department of Neurology, Cedars-Sinai Medical Center, Los Angeles, CA 90048, USA.; 7 Department of Biomedical Sciences, Center for Neural Science and Medicine, Cedars-Sinai Medical Center, Los Angeles, CA 90048, USA.

## Abstract

Humans predominantly explore their environment by moving their eyes. To optimally communicate and process visual information, neural activity needs to be coordinated with the execution of eye movements. We investigated the coordination between visual exploration and interareal neural communication by analyzing local field potentials and single neuron activity in patients with epilepsy. We demonstrated that during the free viewing of images, neural communication between the human amygdala and hippocampus is coordinated with the execution of eye movements. The strength and direction of neural communication and hippocampal saccade-related phase alignment were strongest for fixations that landed on human faces. Our results argue that the state of the human medial temporal lobe network is selectively coordinated with motor behavior. Interareal neural communication was facilitated for social stimuli as indexed by the category of the attended information.

## Teaser

Neural activity across human medial temporal lobe areas is coordinated with saccades and modulated by stimulus category.

## INTRODUCTION

Humans move their eyes to explore their visual environment. During free viewing, saccadic eye movements actively sample visual information by relocating the fovea to target locations. The viewer is unaware of the discontinuities in the visual inputs created by the saccades because the visual system has mechanisms in place to “assemble” the visual scene, fixation by fixation, eliminating the interruptions created by saccades. While we typically do not perceive our own eye movements, the abrupt change in visual input with each saccade has substantial consequences at the neuronal level: A volley of neural activity occurs with each fixation following a saccade in early visual areas (*1*–*6*). Although higher visual areas have larger receptive fields, modulation of neural activity related to eye movements is still prominent (*7*–*9*). Modulation of neuronal activity related to eye movement behavior has been shown even beyond classical visual areas, particularly in the primate medial temporal lobe (*10*–*17*). These studies demonstrate the coordination of neuronal activity and eye movements on a local level and are in line with the notion that vision is an active sensing process intertwining motor sampling routines and sensory perception (*18*–*20*).

Saccadic eye movements disrupt the flow of information three to four times per second (*21*). Thus, multiple neural assemblies across brain areas must be coordinated with the execution of this motor behavior to ensure optimal assembling of visual scenes from sequentially foveated targets. The consequence of this coordination is that the perception of a scene becomes continuous by stitching together the details of the recently foveated areas while also planning future saccades (*22*) to the most salient elements of the visual scene. Different components of the visual scene—e.g., faces, objects, motion, etc.—are processed in parallel by simultaneously active but anatomically distinct areas. A potential mechanism to establish interareal coordination is the alignment of low-frequency oscillatory phase, which has been suggested to reflect the formation of neural assemblies and guide the organization of functional networks on a global level (*23*). Eye movement–related motor signals could serve as a cue to prepare neural assemblies for an incoming volley of neural activity. In other words, the sender and reader of a neural message should be predictively modulated by motor behavior (*24*). Eye movement–related low-frequency phase alignment has been observed in the primate medial temporal lobe (*11*, *16*), and the extent of the phase alignment has been linked to memory performance (*10*, *12*). However, little is known on what the effect of these eye movement–related phase resets is on the state of the brain (as measured by interareal communication), and whether phase resets are conditional on the cognitive significance of the fixated stimulus.

We investigated whether eye movements coordinated neural communication between different components of the human medial temporal lobe by assessing neural interactions between the amygdala and the hippocampus during free viewing of stimuli. Prior studies have shown that neural activity in the amygdala is modulated by the category of visual stimuli viewed by the subject. In particular, many neurons in the human amygdala preferentially fire in response to face or other socially relevant stimuli (*25*–*27*). Strikingly, responses of these face selective neurons are gated by attention, as indexed by fixations and covert attention (*13*). However, the functional significance of these selective visual responses in the amygdala remains unclear. One hypothesis is that these signals are transferred from the amygdala via strong projections (*28*) to the hippocampus, where they elevate and prioritize hippocampal processing of stimuli with high social and emotional significance. This may serve hippocampal memory encoding for salient stimuli and events. In line with this view, enhanced neural communication between the amygdala and the hippocampus has been shown for strongly emotional stimuli (*29*, *30*), but whether this communication depends on eye movements and whether it pertains only to emotional stimuli remain unknown.

We recorded single neuron activity and local field potentials (LFPs) from the human amygdala and hippocampus in conjunction with eye movements, which we took as a measure of attention to stimuli of a specific category, such as faces and objects. We focused our analyses on low-frequency phase interactions since interareal communication is thought to be enabled by the phase of low-frequency oscillations (*23*, *31*). We hypothesized that neural activity in the medial temporal lobe is coordinated with the execution of eye movements and that this coordination is facilitated for prioritized stimuli such as faces. We further hypothesized that in the hippocampus, phase alignment relative to saccade onset depends on stimulus category and will be more prominent for socially relevant faces than for objects.

On the basis of directional connectivity, we confirmed our hypothesis that neural communication between the amygdala and the hippocampus was coordinated by eye movements and modulated by the category of the fixated stimuli. When fixating on faces, the communication between the amygdala and the hippocampus increased as compared to fixating other socially not relevant stimuli. Field-field directionality analyses revealed a unidirectional influence in the network, dependent on the category of the stimuli: Low-frequency activity in the hippocampus was driven by the amygdala when fixating on faces. This saccade-related network alteration was also visible at the local within-brain area level: Saccade-triggered phase alignment was strongest in the hippocampus when fixating face stimuli. Together, these results provide evidence for and suggest that the state of the human medial temporal lobe network is coordinated with motor behavior, and neural communication is facilitated for prioritized stimuli as indexed by the category (here, faces) of the attended information.

## RESULTS

### Behavior and electrophysiology

We analyzed data from 40 recording sessions in 13 patients, comprising in total 1280 channels with LFPs from microwires implanted in the amygdala or hippocampus [which we jointly refer to as the medial temporal lobe (MTL) here]. We isolated 874 single neurons in the MTL (568 in amygdala and 306 in hippocampus). Throughout the manuscript, we use the terms neuron and cell interchangeably to denote an isolated single unit that satisfied specific spike sorting criteria (see experimental procedures for details). All LFP analyses were restricted to recordings from microwires on which we isolated at least one single unit. This assures that the LFPs analyzed were from a high-quality recording sites located in gray matter.

**Fig. 1.**
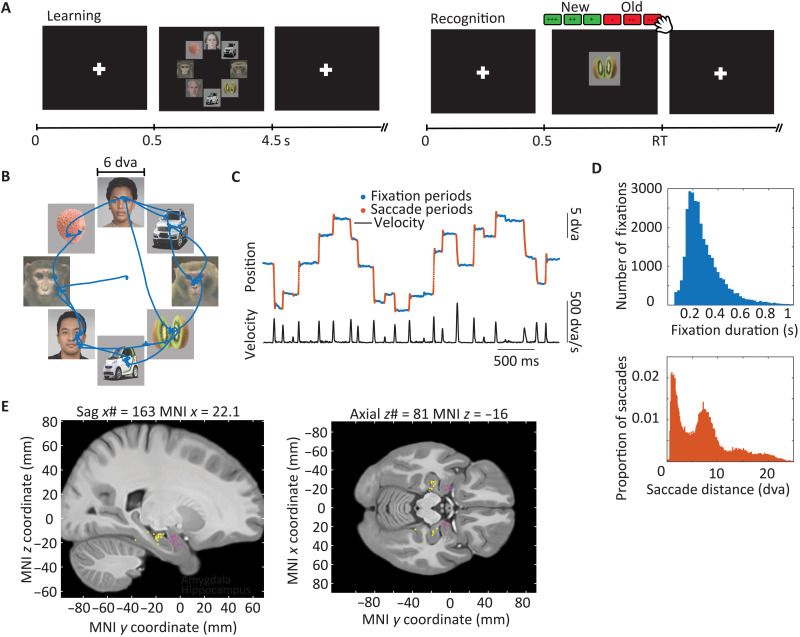
Task, behavior, and recording locations. (**A**) Example of a learning through free-viewing (left) and new/old recognition (right) trial. Subjects report their choice (new versus old) while simultaneously reporting their confidence for that choice. (**B**) Example scan path during free viewing. (**C**) Eye positions and velocity of fixations (blue) and saccades (red) of the free viewing scan path in (B). (**D**) Fixation durations and saccade amplitudes during free viewing. (**E**) Recording sites in the amygdala (pink) and the hippocampus (yellow). Each dot indicates the location of a wire bundle in one patient, projected onto the CIT168 Atlas brain in MNI coordinates.

Patients performed a free viewing task followed by a new/old memory task [Fig. 1, A to D; see (*13*)], during which both eye tracking and electrophysiological data were recorded simultaneously. Stimuli were images picked from four visual categories: human faces, macaque faces, fruits or cars, and flowers or fractals. Previous experiments using the same stimuli showed that human subjects readily explore these images, and neurons in the amygdala respond reliably and preferentially to the human and macaque faces in this stimulus set (*13*). In each trial of the free viewing task, eight stimuli were shown arranged in a circle (Fig. 1A), with two images from each category (two images each were always from the human and macaque face category; the remainder four were from the two nonface categories). Saccades, extracted from eye-tracking data (Fig. 1C), defined the events of interest. Contrasts are based on the category of the fixated stimulus. See fig. S1 for additional behavioral results.

### Familiarity of fixated stimulus modulates latency of responses to human faces in the amygdala

**Fig. 2.**
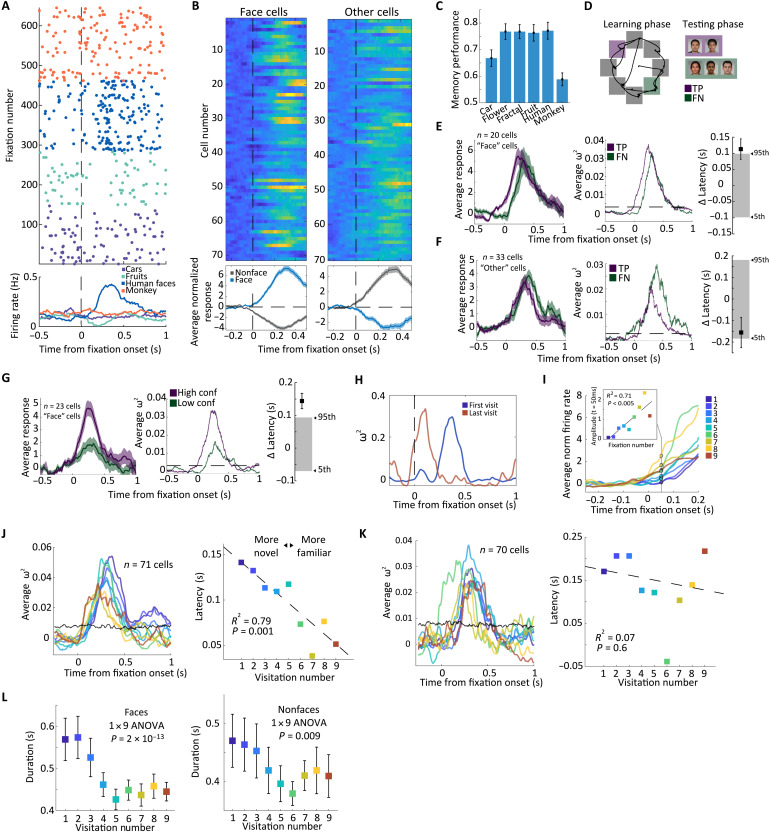
Human face-responsive cells in the human amygdala are modulated by stimulus familiarity during free viewing. (**A**) Example amygdala cell. (**B**) Average normalized activity across all visually selective amygdala cells. (**C**) Memory performance by visual category. (**D**) Grouping of fixations during the learning period into later remembered [TP (true positive)] and not-remembered [FN (false negative)]. (**E** to **G**) Fixation-related response is modulated by familiarity and later memory strength for faces. (E) From left to right, average normalized response, effect size, and latency difference for remembered and not-remembered face stimuli. (F) Same as (E) but for cells tuned to all other visual categories. (G) Same as in (E) but for comparing faces that were correctly recognized with high versus low confidence. (**H**) Effect size for an example cell is shown separately for early fixations (blue) and late fixations (red). (**I**) Normalized response across all amygdala face cells (*n* = 71, measured at *t* = 50 ms) split by visitation number. (**J**) Average image category effect size for the *n* = 71 human face cells, grouped by number of times visited (left). Latency estimate for each group is shown on the left. (**K**) Same as (J) but for cells selective for other stimuli. (I) Behavioral signature of familiarity, as measured by the dwell time on an image. Throughout the figure, face refers to human but not monkey faces.

We first examined whether the response of individual cells during free viewing was modulated by the social relevance and/or the familiarity of the fixated stimuli. This approach is motivated by our prior finding that human faces are disproportionately represented in the human amygdala (*13*), but it remains unknown whether these responses are modulated by stimulus familiarity and whether a similar face preference exists in the hippocampus. While the proportion of visually selective cells (relative to fixation onset) was significantly higher than chance in both the amygdala and the hippocampus [amygdala: 141 of 568, *P* < 0.01; hippocampus: 32 of 306, *P* < 0.01; see fig. S2 (C and D)], the overall proportion of these cells was much larger in the amygdala (25% versus 10%, *P* = 3.7 × 10^−7^). Further, the proportion of all cells that were human face selective was larger in the amygdala versus the hippocampus (12.5% versus 3.3%; 71 of 568 versus 10 of 306 in amygdala and hippocampus, respectively; χ^2^ test of proportions, stat = 20.2, *P* < 1 × 10^−5^). Therefore, while larger than expected by chance (3.3%, *P* < 0.01; see fig. S2D), the proportion of face selective cells was not prominent in the hippocampus. In contrast, human face-selective cells were very common in the amygdala (Fig. 2A shows the fixation-aligned raster of an example face-selective amygdala cell, and Fig. 2B shows the population average). Together, these data show that face-selective responses in the amygdala are more common relative to the hippocampus, indicating a preferential role of the amygdala in detecting the presence of social stimuli. We therefore next examined whether face-related responses in the amygdala are related to stimulus familiarity and memory-encoding success.

After free viewing (32 to 52 learning trials), subjects performed a recognition memory test with confidence ratings (Fig. 1A). Average recognition memory performance was high [71%; 95% confidence interval [0.66 0.76]; Fig. 2C), with some categories easier to remember than others. Recognition memory was higher on high-confidence than low-confidence trials (73% versus 62% for high- and low-confidence trials, *P* = 0.01, two-sample *t* test), as expected for declarative memories (*32*).

We grouped fixations during free viewing into those that landed onto images that were later remembered and those that were later forgotten (Fig. 2D). Comparing the neural response between these two categories of fixations reveals that responses for later-remembered faces appeared earlier than those for faces that were later forgotten (Δ_latency_ = 111 ms, *P* < 0.05 compared to null; Fig. 2E). This effect was specific to neurons that preferred human faces: No similar effect existed for cells that were selective for the other image categories (Δ_latency_ = −156 ms, *P* > 0.05 compared to null; Fig. 2F). To assess further whether this effect was related to memory strength, we next compared the response of human face-selective cells between two different types of remembered trials: those remembered with high and low confidence. This revealed that responses to stimuli later remembered with high confidence appeared significantly earlier (Δ_latency_ = 145 ms, *P* < 0.05 compared to null; Fig. 2G), indicating that response latency is indicative of memory strength.

We next assessed whether responses to fixations on stimuli were modulated by how many times the same stimuli had been fixated before (in earlier learning trials). To do so, we grouped fixations by age, i.e., the number of times that a particular stimulus has been fixated (see Materials and Methods). Behaviorally, the duration of fixations on human faces and other stimuli declined as a function of age, indicating that subjects remembered which stimuli they had looked at before (Fig. 2L). The tuning latency of individual human face cells was correlated with the age of the stimulus, with more familiar stimuli eliciting an earlier response than more novel stimuli (see Fig. 2H and fig. S2F for individual cell examples). Across the 71 human face cells, both the latency of the average normalized firing rate to the faces and the overall tuning (as measured by ω^2^ effect size metric for preferred versus nonpreferred) were modulated by the age of the fixation (Fig. 2, I to J). This effect was only present for face-selective cells (Fig. 2K). As a control, we also repeated this analysis after eliminating cells selective for stimuli with lower memory performance (“cars” and “monkeys”). This effect was unchanged (fig. S2G), showing that memory strength cannot explain the difference between human face and other-stimuli tuned cells.

### Saccade-related field-field connectivity between amygdala and hippocampus

**Fig. 3.**
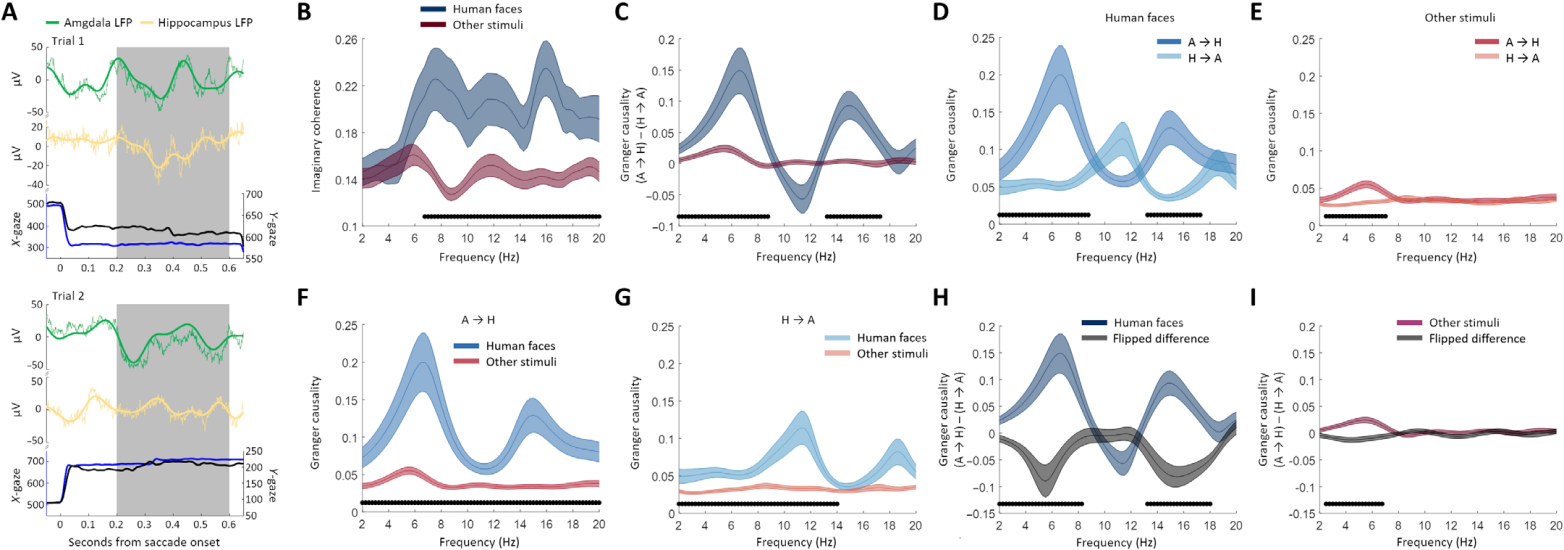
Saccade-related neural connectivity between amygdala and hippocampus is modulated by the stimulus category faces. (**A**) Example single trials for simultaneously recorded amygdala (green) and hippocampus (yellow) LFPs (gray indicates time window of interest; thick line: filtered data; *T* = 0 is saccade onset). (**B**) Increased amygdala-hippocampus iCoh for human faces versus other stimuli. (**C**) GC differences are larger when fixating human faces compared to fixating other stimuli (peaks: 6.5 and 15 Hz). (**D**) GC when fixating human faces indicates that amygdala drives hippocampus (peaks: 6.5 and 15 Hz). (**E**) GC when fixating other stimuli indicates that amygdala drives hippocampus but to a lesser extent (peak: 5.5 Hz). (**F**) A → H GC is larger when fixating human faces (peaks: 6.5 and 15 Hz). (**G**) H → A GC is larger when fixating human faces (peak: 11 Hz) (F and G). (**H** and **I**) Flipping the time series reversed directions of GC interactions (H) and GC difference (A → H minus H → A) for fixating human faces. (I) GC difference (A → H minus H → A) for fixating other stimuli. Shading depicts SEM. Black dots indicate contiguous frequency bins showing significant differences (corrected for multiple comparisons).

We next determined whether neural communication assessed via amygdala-hippocampus field-field (“LFP-LFP”) interactions was coordinated with eye movements and whether this coordination was also modulated by the category of the fixated stimuli (see fig. S5, for example, spectra of the LFP). To answer these questions, we contrasted the extent of neural connectivity between two conditions: (i) fixating on human faces and (ii) fixating on other stimuli (Fig. 1A). All analyses were performed in the post-saccadic interval that follows saccade onset (Fig. 3A). We used two methods to assess neural interactions: frequency resolved imaginary coherence (iCoh) (*33*), which removes potential volume conduction effects by eliminating zero-phase lag coupling, and Granger causality (GC) (*34*, *35*), which allows an assessment of directionality. We used data from 200 to 600 ms following saccade onset for analysis (Fig. 3A). The 200 ms immediately after saccade onset was not included to account for visually evoked and saccadic spike potentials (*10*). Also, to avoid potential artifacts from other eye movements, only trials that were free of saccades and blinks in this post-saccadic time interval were included (*n* = 342 trials were included; see Materials and Methods). Only those saccades followed by a fixation falling on one of the presented images were included in the analyses.

### Coherence between amygdala and hippocampus is directional and modulated by the category of the attended stimulus

We found significantly increased iCoh between amygdala and hippocampus for human faces compared to other stimuli from 6.5 to 20 Hz (*P* < 0.001; Fig. 3B), with peaks at 7.5, 11.5, and 16 Hz. Thus, when participants fixated on human faces, neural communication (field-field coherence) between amygdala and hippocampus was increased as compared to when they fixated on other stimuli.

Was this interaction directional? To assess this question, we used GC to quantify the direction of information flow between amygdala and hippocampus LFPs in the frequency domain. GC analysis relies on the comparison of a model that predicts a time series *x* (e.g., hippocampal LFPs) based on past values of *x* to a bivariate model that predicts *x* based on past values of *x* and past values of a different time series *y* (e.g., amygdalar LFPs). We found that gaze-related GC was modulated by the category of the attended stimulus (Fig. 3, C to I). The amygdala exerted a significantly stronger GC influence on the hippocampus when human faces were fixated (Fig. 3C) compared to when other stimuli were fixated, with two significant clusters peaking at 6.5 Hz (*P* < 0.005) and 15 Hz (*P* < 0.05). Post hoc tests confirmed that the GC influence from amygdala on the hippocampus was higher than vice versa (hippocampus on amygdala) for human faces (*P* < 0.0005; Fig. 3D) and for other stimuli (peaking at 5.5 Hz, *P* < 0.005; Fig. 3E). The GC influence of one area on the other was significantly stronger when fixating on human faces compared to other stimuli. This was true for both the influence of the amygdala on the hippocampus (*P* < 0.0005; Fig. 3F) and the influence of the hippocampus on the amygdala (*P* < 0.005, peaking at 11.25 Hz; Fig. 3G).

As a control for potential confounds due to the sensitivity of GC to fluctuations in noise levels, we next analyzed GC in time-reversed data (*36*). A true GC influence between two signals (reflected as *y* Granger causes *x*) that is time-reversed should lead to a reversal of GC (*x* Granger causes *y*). In contrast, time-reversing a spurious interaction should not result in reversal of GC but instead show a similar GC directionality because time reversal does not affect the local noise level. We find that time-reversing our data results in a reversal of the GC patterns (Fig. 3, H and I). We can thus exclude that our effects were driven by differences in noise levels between the two time series. As a second control, we also assessed whether our GC analysis was influenced by condition-specific differences in LFP power. However, we found that LFP power between the conditions was not significantly different around the peaks in the GC analyses (see fig. S3), also alleviating this concern. As a third control, we assessed whether our main effects (amygdala drives hippocampus) are sensitive to signal-to-noise differences between regions. To do so, we excluded the 10% of trials with the largest power difference (amygdala minus hippocampus). Patterns of amygdala-hippocampus interactions were highly similar after this exclusion (see fig. S4), alleviating this concern. Last, we also computed iCoh and GC before saccade onset and compared them to our results (which are computed post-saccade). This revealed that post-saccade iCoh and GC were significantly larger than pre-saccade iCoh and GC (see fig. S9), supporting our argument that our results are due to post-saccadic modulation.

To assess whether iCoh and GC interactions were specific to within-hemisphere interactions (only unilateral pairs were used for analyses so far), we computed iCoh and GC for contralateral amygdala-hippocampus pairs. Contralateral iCoh and GC differed substantially from our main results (see fig. S6), with no robust differences in directional interactions between faces and other stimuli. This result indicates that information flow from the amygdala to the hippocampus is only coordinated within the same hemisphere (see Discussion).

Behavioral analysis revealed several key differences between the human face and other stimuli categories. We therefore performed several control analyses to exclude the possibility that these differences could explain our results (rather than the human face versus others contrast). First, some subcategories (monkey faces and cars) were harder to remember than faces (see Fig. 2C). After excluding these subcategories from the “other stimuli” (thereby resulting in equal memory strength; see Fig. 2C), our results remain qualitatively identical (see fig. S7). This indicates that the connectivity patterns we found were not due to differences in memory strength for the different subcategories. Second, the total number of fixations made to faces was larger than to other stimuli (see fig. S1B). We therefore also compared GC and iCoh by splitting other stimuli trials based on the total number of fixations. The results did not reveal patterns of interaction similar to our principle findings (see fig. S8), therefore indicating that our result was not driven by differences in fixation frequency.

### Category-dependent modulation of saccade-related LFP phase alignment

Next, we asked whether saccade-related local neural activity was modulated by the category of the fixated stimulus. LFP activity has been shown to be temporally aligned with the execution of saccadic eye movements in some brain areas and species (*10*–*14*, *16*), but no systematic comparison between the extent of these effects in the human amygdala and hippocampus and their relationship to the identity of the fixated stimuli exists. We here investigated where in the medial temporal lobe a saccade-related phase alignment of LFP could be identified and whether a potential phase alignment would be modulated by the category of the attended stimulus. A phase alignment index (PAI) was computed to quantify saccade-related phase alignment within the amygdala and the hippocampus. PAI is the difference between the pre-saccadic and the post-saccadic ratio of trial-averaged LFP power and single-trial LFP power. A positive PAI indicates an increase of phase alignment in the post-saccadic intervals relative to the pre-saccadic interval. Frequency-resolved PAI was computed for each condition and area, respectively. Post-saccadic intervals included data points from 200 to 600 ms after saccade onset. Pre-saccadic intervals included data points from 400 ms to saccade onset. The 200 ms immediately after saccade onset were not included to account for visually evoked potentials and saccadic spike potentials. Also, to avoid potential artifacts from other eye movements, only trials that were free of saccades and blinks in the respective pre- and/or post-saccadic interval were included. Only those saccades followed by a fixation falling on one of the presented images were included in the analyses (*n* = 733 trials were included; see Materials and Methods).

**Fig. 4.**
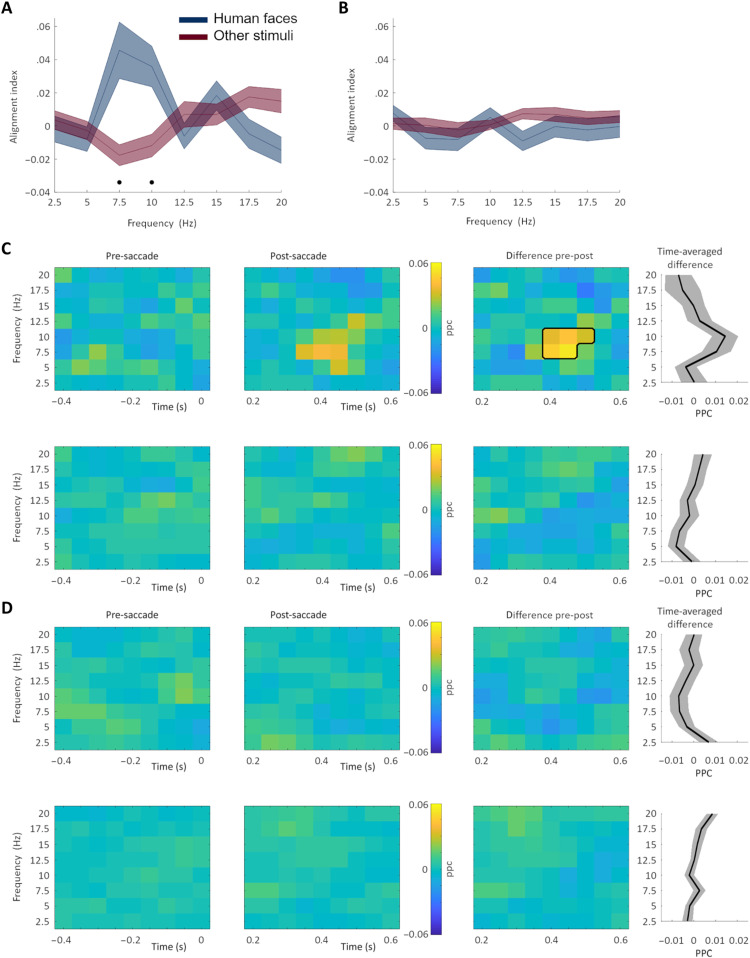
Category-dependent modulation of saccade-related phase alignment. (**A**) Hippocampal saccade–related phase alignment is significantly increased for fixations on human faces versus fixations on other stimuli at 7.5 to 10 Hz. (**B**) Amygdala saccade–related phase alignment shows no significant difference between conditions. Shading depicts SEM. Black dots indicate frequency bins showing significant differences between conditions (corrected for multiple comparisons across frequencies). (**C** and **D**) Category-dependent modulation of saccade-related PPC. (C) Hippocampal saccade–related PPC is increased at 7.5 to 10 Hz when comparing pre- to post-saccade periods (top row; black outline indicates significant difference). No obvious increase from pre- to post-saccade is present when fixating on other stimuli (bottom row). (D) Amygdala saccade–related PPC shows no obvious differences between pre- to post-saccade periods in both conditions (human faces, top row; other stimuli, bottom row).

Comparing the PAI across conditions (human faces versus other stimuli), areas (amygdala and hippocampus), and frequencies (2.5 to 20 Hz, in 2.5-Hz steps) with a three-way analysis of variance (ANOVA) revealed a significant “condition × frequency × region” interaction (*F*_7,115_ = 5.86, *P* = 0.000001). We then compared the PAI between conditions in each area (while controlling for multiple comparisons across frequencies) and found significantly higher PAI values in the hippocampus for fixations on human faces versus other stimuli at 7.5 to 10 Hz (*P* < 0.0032; Fig. 4A). There was no significant difference in the PAI between conditions in the amygdala (*P* > 0.14; Fig. 4B).

To further evaluate saccade-related phase alignment, we next computed pairwise phase consistency (PPC) (*37*) in the pre- and post-saccadic time window as a function of time and frequency. In the hippocampus, PPC increased for post-saccadic periods as compared to pre-saccadic periods at 7.5 to 10 Hz (*P* < 0.02, cluster-corrected) but only when the fixation fell on human faces (Fig. 4C). No obvious differences in amygdala PPC were found between pre- and post-saccadic periods, neither when fixations fell on faces nor when they fell on other stimuli (Fig. 4D). Thus, the PPC results are in line with the results of the PAI analyses, pointing toward a category-dependent modulation of saccade-related phase alignment specifically in the hippocampus. As a control for the temporal relationship between saccade onsets and PAI, we analyzed PAI locked to fixation onsets. No significant category-dependent modulation of PAI was found when relating signals to fixation onsets (see fig. S10). In line with our PAI results, single-trial LFP power did not differ between pre- and post-saccade time windows for the frequencies of interest (7.5 to 10 Hz; *P*’s > 0.1), further alleviating concerns about single-trial power being the driver of the observed PPC effects.

Together, the PAI and PPC results indicate that there is a category-dependent modulation of saccade-related phase alignment in the hippocampus, but not the amygdala. When fixations fell on human faces, hippocampal post-saccadic phase were aligned at 7.5 to 10 Hz, a frequency range compatible with what we found by analyzing interareal interactions.

## DISCUSSION

Our results reveal that neural activity in two key structures of the human medial temporal lobe is coordinated with visual exploration behavior and prioritizes socially relevant stimuli (human faces). We report here that interareal neural synchronization between the amygdala and the hippocampus is coordinated with eye movements. Expanding previous studies that show coordination between eye movements and local neuronal activity (*10*–*17*), we show that this coordination spans across multiple areas involved in visual exploration. These findings bring further evidence in favor of the notion that vision is an active sensing process linking motor behavior and sensory-perceptual processing (*18*–*20*).

At the single neuron level, the proportion of cells that were visually selective for human faces (as well as other stimuli) following fixation onset was substantially larger in the amygdala relative to the hippocampus. This finding indicates that the amygdala plays a more important role in detecting the presence of socially meaningful stimuli (*27*). This finding also shows that, as in trial-based analysis (*38*, *39*), the proportion of visually selective cells is higher in the amygdala relative to the hippocampus when analyzed fixation-by-fixation. The time at which the face-selective (but not other) responses first appeared relative to fixation onset in the amygdala was modulated by stimulus familiarity and was predictive of the strength and success of memory encoding. This indicates that the face-evoked responses in the amygdala are modulated both by saccades and by memory, indicating that they reflect input from both processes.

A key finding is that saccade-related interareal communication was modulated by stimulus category. When a fixation on a human face followed a saccade, neural communication between the amygdala and hippocampus was enhanced. The same effect was not observed for saccades and fixations that landed on other stimuli, indicating that interareal communication can be prioritized or amplified for attending stimuli of high social relevance (human faces). Our results show that hippocampus-amygdala field-field interactions that were first reported for strongly emotional stimuli (*29*, *30*) are present for relatively emotionally neutral stimuli that carry high social significance and are modulated by eye movements.

The directionality of the coherent activity in the amygdala and the hippocampus revealed important details on how socioemotional relevance, extracted in the amygdala, may modulate memory encoding in the hippocampus. We show that the amygdala drives hippocampal activity, particularly when saccades land on a human face, i.e., signals from the amygdala alter mesoscale population activity (indexed by LFP) in the hippocampus. At first sight, this might be at contrast with previous studies in rodents that showed hippocampal theta oscillations entraining neuronal firing in the amygdala firing (*40*, *41*) or bidirectional modulations of human amygdala-hippocampus field-field interactions when viewing strongly emotional stimuli (*29*). The finding relating to theta modulation might be related to species-specific differences. The human amygdala plays a critical role in face processing (*25*, *26*, *42*), and also humans explore and extract important socioemotional information from faces by foveating specific facial features such as the eyes. The neurophysiological correlates of these functions may not exist or have not been characterized yet in rodents where the hippocampal activity has been shown to drive the amygdala. Comparable, bidirectional interactions between the human amygdala and hippocampus have been documented in the theta frequency band (*30*), but these may be related to memory-demanding pattern separation tasks that might activate the hippocampus more than our novel-familiar discrimination task. Here, we replicated previous findings that, in the human amygdala, neuronal activity is gated by eye movements (*13*), and this activity, in turn, prepares hippocampal processes for high-priority input arriving with the next fixation. This may explain the predominant drive of the hippocampus by the amygdala at a frequency of 6 to 7 Hz as indexed by GC. Accordingly, the human amygdala is upstream of the hippocampus during tasks of identifying categories of stimuli. Direct subcortical pathways from early sensory areas to the amygdala might enable rapid assessment of information content (*43*, *44*). The face conditional information flow between the amygdala and hippocampus that we found was present only ipsilaterally, but not across hemispheres. This indicates that processing of social stimuli occurred largely independently between hemispheres compatible with the notion that anterior hippocampi in humans act largely independently due to the absence of the ventral hippocampal commissure (*45*, *46*). The same functional independence can be inferred for the left and right amygdalae from the anatomical literature. In both humans and non-human primates, the amygdalae are only weakly connected through the anterior commissure (*47*, *48*). Together, our results support the notion that the oscillatory phase sets the stage for information transfer between brain regions in line with current frameworks of neural communication (*24*, *49*).

The strength of saccade-related phase alignment depended on the stimulus: In the hippocampus, it was present only when a saccade landed on a human face. This finding confirms previous reports (*10*, *11*, *17*) and, in addition, shows that this phase alignment is not a pure reflection of the execution of the motor movement. Rather, it is conditional on the content of the to-be fixated stimulus that the saccade targets, thereby revealing that saccade-related phase alignment in the hippocampus reflects the result of high-level cognitive processing in addition to a motor command. In line with previous findings, the observed differences were related to saccade onset rather than fixation onset, indicating that the hippocampal phase was aligned to the execution of motor commands. While the precise pathways of motor commands (or, rather, their copies, i.e. a corollary discharge) to the human medial temporal lobe are not known, a prime candidate would be a route via brainstem and thalamus (*50*). What function could a hippocampal phase alignment serve? The hippocampus is assumed to have duty cycles for encoding versus retrieving memories reflected by opposing phases in the hippocampal LFP (*51*–*53*). Accordingly, the here described hippocampal phase reset could reflect a switching of the hippocampal duty cycle to facilitate encoding of the prioritized face stimuli.

Our results highlight the importance of coordination between saccadic eye movements and low-frequency activity in the human medial temporal lobe. Low-frequency oscillations, particularly hippocampal theta, are less continuous in primates than in rodents (*54*, *55*). The present results might help to reconcile the seemingly bursty nature of human low-frequency oscillations by explaining parts of the intermittent rhythmicity through the functional coordination between brain rhythms and eye movements. If, for example, the hippocampal phase is aligned to saccades, and the strength of alignment depends on the stimulus category, this would disrupt potentially continuous oscillations. While our paradigm encouraged visual exploration through large and frequent eye movements, smaller eye movements are also present during periods of fixation (*56*, *57*). Whether these smaller fixational eye movements are also coordinated with neural activity remains an open question. We note that a challenge for analysis of saccade-related neural processes during unrestrained free viewing behavior is that only the subset of saccades that are followed by relatively long fixations can be analyzed. This is because this is the only way to disentangle processes triggered by the current saccade from those triggered by later saccades. An open question raised by our results is whether similar processes are also apparent during shorter fixations and, if so, how these processes are superimposed on top of each other for rapid sequences of saccades. Note that the restrictions on saccade selection with respect to fixation duration together with a low number of forgotten trials impeded analyzing subsequent memory contrasts for the connectivity metrics. Future studies could address this question in a paradigm with optimally balanced remembered and forgotten trials.

Field-field interactions from amygdala to hippocampus were strongest at 6.5 Hz. Hippocampal influence on amygdala, on the other hand, peaked at 11 Hz and was smaller in magnitude than the amygdala to hippocampus interaction. The finding that interactions of different directions can occur at different peak frequencies indicates that multiple neural assemblies might establish different channels for directional communication between brain regions (*49*). For example, in the visual system, feedforward and feedback interactions are thought to be subserved by different frequencies (*58*–*60*). While less is known about interactions within the MTL, our data indicate that a similar mechanism might be used to coordinate information flow between the amygdala and hippocampus. The non-human primate amygdala contains multiple putative subnetworks indexed by distinct neural coactivity patterns that are characterized by different dominant frequencies in 0- to 20-Hz range (*61*).

In conclusion, we here present evidence that neural activity and eye movements are coordinated on a network level and that the communication within the network can be facilitated for prioritized stimuli (here, socially relevant human faces), affecting local processing in downstream areas. Our results support the view that neural assemblies across brain areas are coordinated with the execution of motor behavior (*18*–*20*) to optimally communicate and process foveated stimuli. Phase-based interareal communication supports the notion that the LFP phase guides the organization of functional networks across brain regions and mediates information transfer (*23*, *24*, *31*). The state of the network communication depends on the category of the stimuli processed, with socially relevant stimuli being prioritized. The consequence of this prioritized communication could be observed in a saccade-related hippocampal phase alignment that was elevated for face stimuli. Considering the specialization of the amygdala for processing socially relevant stimuli (*13*, *25*, *26*), it is reasonable to assume that the amygdala signals the arrival of relevant visual information to the hippocampus, which, in turn, prepares to optimally and selectively process the input.

## MATERIALS AND METHODS

### Experimental design

The patients were instructed to freely view complex visual stimuli for a later memory test [Fig. 1A; see (*13*)]. Each stimulus consisted of a circular array of eight images randomly chosen from two face categories (human and monkey faces) and two nonface categories (either flowers and fractals or fruits and cars, depending on the version of the task performed). Each image array was displayed for 4 to 6 s, and subjects were free to view any location. After each array, a fixation cross was displayed for 1 s before the next trial began. Patients viewed 48 to 52 array trials followed by 48 single image trials for the memory test. During the memory test, subjects reported their choice and confidence simultaneously using a button box with six options; three buttons corresponded to low-, medium-, and high-confidence “old” trials, and three corresponded to low, medium, and high confidence for “new” trials. The task was implemented in MATLAB using the Psychophysics Toolbox (*62*). Note that the human faces shown in Figs. 1 and 2 and fig. S2 do not correspond to real people but were generated using a pretrained generative adversarial network. We used these images to protect the identity of the people whose faces were actually used in the experiment.

### Participants

Thirteen patients who were being evaluated for surgical treatment of drug-resistant epilepsy provided informed consent and volunteered for this study. The Institutional Review Boards of Cedars-Sinai Medical Center and the California Institute of Technology approved all protocols.

### Electrophysiology

We analyzed data from 13 patients recorded in 40 sessions comprising 1280 LFPs. Of these 40 sessions, we excluded four sessions where the eyes of the human and monkey stimuli were occluded. Therefore, our analyses are based on the remaining 36 sessions and 1152 electrodes where we recorded LFPs. We recorded bilaterally from the hippocampus and the amygdala using microwires embedded in macroelectrodes. From each microwire, we recorded the broadband 0.1- to 9000-Hz continuous extracellular signal with a sampling rate of 32 kHz (Neuralynx Inc.). One microwire on each macroelectrode served as a local reference (bipolar recording), except for three sessions that were not locally referenced during the recordings. In these three sessions, LFP signals were rereferenced to the common average across all micro wires embedded in a macroelectrode. From the microwires, we isolated 874 single neurons in the MTL (568 in amygdala and 306 in hippocampus). Throughout the manuscript, we use the terms neuron or cell to refer to a putative single unit, and we used only units satisfying multiple conservative criteria (see Experimental Procedures for details). All LFP analyses were restricted to microwires with at least one single unit. A subset of the dataset analyzed here (amygdala data from 28 sessions) is further analyzed in (*13*).

### Spike sorting

The raw signal was filtered with a zero-phase lag filter in the 300- to 3000-Hz band, and spikes were detected and sorted using a semiautomated template-matching algorithm (*63*, *64*).

### Localization of electrodes

Electrodes were localized on the basis of pre-T1 structural magnetic resonance imagings (MRIs) and postoperative MRI and/or computed tomography scans. We coregistered all electrode locations onto an atlas brain as previously described (*13*). Only electrodes that could be localized to the amygdala or hippocampus were included.

### Eye tracking and trial definition

Monocular gaze position was monitored at 500 Hz (EyeLink 1000, SR Research). Calibration was performed using the built-in nine-point calibration grid and was only used if validation resulted in a measurement error of <1 dva (average validation error was 0.62 dva). We used the EyeLink system, automatic annotation of fixations and saccades from the continuous stream of data using a motion, velocity, and acceleration threshold (default thresholds). Each onset of a saccade, extracted from eye-tracking data, defined an event of interest for the saccade-related analyses. To investigate the synchronization between LFPs (see below), post-saccade intervals of 400-ms length were analyzed. Post-saccade intervals included data points ranging from 200 to 600 ms after saccade onset. For the phase alignment analyses (see below), post-saccade and pre-saccade intervals were analyzed. Pre-saccade intervals started at 400 ms before saccade onset and led up to saccade onset. The 200 ms immediately after saccade onset was not included in any analyses to account for visually evoked potentials and saccadic spike potentials. To avoid potential artifacts from other eye movements, only trials that were free of saccades and blinks in the respective pre- and/or post-saccade interval were included. For all saccade-related analyses, only those saccades followed by a fixation falling on one of the presented images were included in the analyses.

Contrasts were based on the category of the fixated stimulus (human faces versus other stimuli; other stimuli included flowers, fractals, fruits, cars, and monkey faces). Only microwires with at least 15 trials per condition entered the analyses (that is, sessions that contributed less than 15 trials for each condition were excluded). On the basis of these exclusion criteria, different overall trial numbers (see below) resulted for local (PAI and PPC) versus connectivity (iCoh and GC) metrics. While it is, for example, sufficient that there are no artifacts in the one region (amygdala or hippocampus) where PAI and PPC is computed in any given trial, it is necessary that there are no artifacts in both regions for any given trial when computing iCoh and GC.

### Single neuron analysis

#### Cell selection

Each cell was classified as visually selective if its firing rate in the 500 ms after fixation onset was modulated by the category of the fixated image, as determined by a 1 × 4 ANOVA. The preferred stimulus for each visually selective cell was set to the image category for which the firing rate was the greatest in the selection window. To control for differences in viewing time across categories, we treated fixation duration as a nuisance regressor and computed the visual category ANOVA on the residual firing rate. Firing rate over time was computed using a 250-ms moving window, with a 1-ms step size, and smoothed with a 10-ms Gaussian kernel. The proportion of selected cells was compared to a null distribution, which was constructed by repeating the selection procedure 1000 times with shuffled visual category labels across all fixations.

#### Fixation selection

In each session, subjects generated hundreds of fixations (923 ± 301 fixations). We were selective in which subset of these fixations we included in our analysis to control for possible biases that might arise from the subjects’ sampling biases. The inclusion criteria for fixation selection were as follows: (1) We only use the first fixation that lands on a stimulus with successive fixations within that stimulus being ignored, (2) average dwell time on a stimulus must be greater than 100 ms, (3) only data from the learning portion of the experiment is considered, and (4) fixations on an image that is of the same visual category as the preceding image (ex. face-to-face transition) are removed from the analysis. See fig. S2B for an example of fixation selection in a learning trial. The main reason for criterion (4) is to diminish the effect of neural activity that carries over from the previous stimulus.

#### Effect size

We use the omega-squared (**ω**^2^) effect size metric to evaluate the strength of tuning for the preferred versus nonpreferred visual category. Omega-squared is defined as follows

$\omega^{2}=\frac{{\mathrm{SS}}_{\text{category}}-{\mathrm{df}}_{\text{category}}\cdot {\mathrm{MS}}_{\text{error}}}{{\mathrm{SS}}_{\text{total}}+{\mathrm{MS}}_{\text{error}}}$

#### Visitation number

With the exception of the very first trial, each array of images (there are 32 to 48 arrays during the learning phase) contains a combination of stimuli that have been shown previously in the experiment and novel images (i.e., never shown before) (Fig. 2, H to L, and fig. S2, D, F, and G). We label each fixation on a stimulus with its age value, defined as the number of times the subject has visited that stimulus since the beginning of the experiment. Age values for fixations vary broadly within a session, ranging from 1 (this is the first fixation on this image) to >30. Age values also vary across sessions, reflecting the subjects’ idiosyncratic viewing preferences. To align across sessions, we discretize the age values within a session into nine bins, the edges of which are computed using percentiles over the entire distribution of age values within that session. The nine colors in Fig. 2 (I to L) correspond to these bins. Note that for a given cell, because of the fixation selection procedure, one of these bins might be empty. For example, it might happen that all fixations in the seventh bin are <100 ms in duration and therefore are all invalid.

#### Memory and confidence contrast

We use the subject’s performance during the recognition phase to split fixations during the learning phase into groups (Fig. 2, E to G, and fig. S2E). The first contrast is between remembered and not-remembered faces. The second contrast is between faces that were correctly remembered with high and low confidence. Note that while there are 71 “face cells” and 70 “other cells,” these analyses were performed on a subset of the cells. The reason for this is that not all sessions had incorrect trials since performance on target trials (as opposed to the lure trials) was high. To equalize group sizes, we subsampled trials from the larger group to match the smaller one (the smaller groups across cells are “incorrect” and “low confidence”). As a control, we confirmed that results are qualitatively similar by using a matching procedure that only includes cells if they contribute to all groups (fig. S2E).

#### Latency estimation

To compute effect size latency for a given condition, we generate a null distribution by shuffling the preferred/non-preferred label of the visual stimuli (Fig. 2, E to G and J to K, and fig. S2F). The latency is then defined as the point where the true effect size crosses the 99th percentile of the null distribution. In some of the figures (e.g., Fig. 2E), the threshold is indicated with a dotted black line. To compute statistics for the difference in latency between conditions [e.g., between true positive (TP) and false negative (FN) in Fig. 2E], we generate a null distribution by shuffling the condition label of the effect size traces across cells. To illustrate the procedure, we consider the latency comparison between the TP and FN conditions in Fig. 2E. Each of these conditions is made up of 20 traces (one for each cell). To generate the null distribution in this case, we shuffle the TP/FN labels for each trace and, for each iteration, compute a latency difference. We repeat this procedure 1000 times to create the null distribution.

### LFP preprocessing

Artifacts were identified via automatic detection (excessive amplitudes) and visual inspection (e.g., epileptiform spikes). Contaminated epochs were excluded from the analyses. Spike-related transients were removed from LFP traces using linear interpolation.

### Spectral analysis

The frequency spectra for phase and power were computed by applying a Fourier transformation to the 400 ms of the data in the pre- and post-saccade time windows after multiplying each time window with a hanning taper. Accordingly, phase and power were estimated in steps of 2.5 Hz for frequencies between 2.5 and 20 Hz. To depict the temporal dynamics of the saccade-related phase alignment, a sliding time window with a window length of 0.4 s and steps of 0.05 s was used to compute time-frequency representations.

### Synchronization between LFPs

To investigate gaze-related neural communication between medial temporal lobe LFP signals, we computed iCoh and GC in the frequency domain. A total of 342 trials (117 for human faces and 225 for other stimuli) were included in these analyses.

#### Imaginary coherence

We computed iCoh (*33*) between unilateral pairs of amygdala and hippocampus LFP recordings locked to saccade onsets to investigate gaze-related neural communication among medial temporal lobe areas. iCoh is obtained by projecting the complex-valued coherency values onto the imaginary axis, eliminating instantaneous interactions between pairs of LFP recordings, thereby removing potential contributions of volume conduction to the estimation of neural synchronization.

#### Granger causality

GC analysis (*35*, *65*) was used to investigate the direction of information flow between gaze-related LFPs in the hippocampus and the amygdala (unilateral pairs only). GC analysis relies on the comparison of a univariate model that predicts the future of a time series *x* based on past values of *x* to a bivariate model that predicts the future of an *x* based on past values of *x* plus past values of a different time series *y*. If the variance of the prediction error is smaller in the latter than the former model, then *y* is assumed to have a Granger causal influence on *x*. Here, GC was calculated nonparametrically in the frequency domain between pairs of LFP recordings (*66*, *67*). To this end, the noise covariance and transfer function are computed from the spectral density matrix [as implemented in fieldtrip; (*68*)] based on the Fourier coefficients. The nonparametric frequency domain version of GC does not require the determination of the model order for the autoregressive model.

GC can be sensitive to differences in signal-to-noise ratios between two time series, potentially causing spurious differences in directionality. To diagnose this, GC was also computed for time-reversed signals and compared to the actual nonreversed GC (*36*). This strategy was adopted because a true, causal influence between two signals (reflected as *y* Granger causes *x*) that is time-reversed leads to a reversal of GC (*x* Granger causes *y*), whereas time-reversing a spurious interaction should not result in reversal of GC.

### Phase alignment

To investigate saccade-related phase alignment, a PAI was computed. The ratio of trial-averaged power (i.e., averaging trials and estimating the power on the event-related average) and single-trial power (i.e., estimating power for each single trial and then averaging across trials) was computed for pre-saccadic and post-saccadic time windows. The difference between the two ratios constitutes the PAI. A positive PAI can be interpreted as an increase of phase alignment from pre- to post-saccadic intervals. To further evaluate the saccade-related phase alignment, PPC (*37*) was computed in the pre- and post-saccadic time window, respectively. A total of 733 trials (253 for human faces and 480 for other stimuli) were included in these analyses.

### Controlling for unequal trial numbers

Because iCoh, GC, PAI, and PPC can vary as a function of the number of trials (defined by either number of saccades, number of spikes, or number of stimuli per condition) used in the calculation, we equalized the number of trials in the two groups (human faces versus other stimuli) by randomly selecting a subsample of trials from the larger group. The subsample’s number of trials was equal to the smaller group’s number of trials. iCoh, GC, PAI, and PPC in the larger group were calculated as an average of 500 repetitions of random subsample selections.

### Statistics

Differences in PAI, iCoh, and GC between the two conditions were compared by means of a cluster-based nonparametric permutation test (*69*) controlling for multiple comparisons in the frequency domain. Continuous frequency clusters with significant differences between human faces and other stimuli were identified by computing paired sample *t* tests (*P* < 0.05, two-tailed) across microwire recordings. The cluster-level statistics was defined as the sum of the *t* values in a given cluster and tested against the permutation distribution (Monte-Carlo method, 5000 randomizations). The null hypothesis that there was no difference between conditions was rejected at an alpha level of 0.05 (two tailed). Note that, by design, the cluster-based permutation tests we used indicate ranges of frequencies in which conditions are significantly different (i.e., 5 to 12 Hz), but not at what frequency that difference was maximal. We used the grand average to identify the frequency at which the difference was maximal. However, note that all statistics are based on the entire range and not the peak frequency identified this way.

Differences in PPC between the two conditions in the hippocampus were compared by means of a cluster-based nonparametric permutation test (*69*) controlling for multiple comparisons in the time and frequency domains. Continuous time-frequency clusters with significant differences between human faces and other stimuli were identified by computing paired sample *t* tests (*P* > 0.05, two tailed) across microwire recordings. The cluster-level statistics was defined as the sum of the *t* values in a given cluster and tested against the permutation distribution (Monte-Carlo method, 5000 randomizations). The null hypothesis that there was no difference between conditions was rejected at an alpha level of 0.05 (two tailed).

Statistical analyses were performed at the individual electrode (or electrode-pair) level (fixed-effect analysis), considering all electrodes/pairs that were eligible based on our criteria (e.g., minimum number of trials, etc.).

## Notes

View/request a protocol for this paper from *Bio-protocol*.
