# Supplementary material for: Saccade-related neural communication in the human medial temporal lobe is modulated by the social relevance of stimuli
Source: Sci Adv. 2022 Mar 18;8(11):eabl6037. doi: 10.1126/sciadv.abl6037 (PMC8932656; doi:10.1126/sciadv.abl6037)
Supplement: Supplementary file 1 — Figs. S1 to S10 [file sciadv.abl6037_sm.pdf]

Science Advances
AAAS

Supplementary Materials for

Saccade-related neural communication in the human medial temporal lobe is modulated by the social relevance of stimuli

Tobias Staudigl, Juri Minxha, Adam N. Mamelak, Katalin M. Gothard, Ueli Rutishauser*

*Corresponding author. Email: ueli.rutishauser@cshs.org

Published 18 March 2022, *Sci. Adv.* **8**, eabl6037 (2022)
DOI: 10.1126/sciadv.abl6037

This PDF file includes:

Figs. S1 to S10

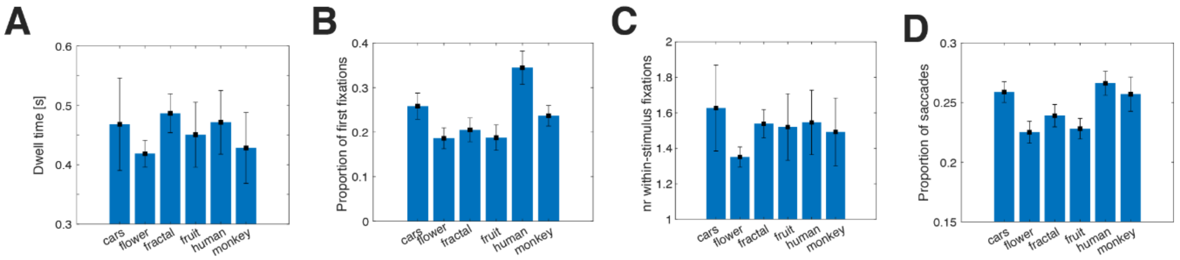

Figure S1. Behavioral controls

(A) Average dwell time (i.e., time spent looking at one image) as a function of image category (0.47s vs. 0.43s for human vs. monkey respectively; $p = 0.001$, paired sample t-test). (B) Proportion of first fixations landing on each of the image categories. The location of different image categories is randomized across trials so this reflects a clear attentional preference for human faces (0.35 vs. 0.24 for human and monkey respectively; $p = 8.2e-5$, paired sample t-test). (C) Average number of *within*-stimulus fixations (ex., subjects might look at the eyes before making a saccade to the mouth region of a face) as a function of image category (1.55 vs. 1.49 for human vs. monkey respectively; $p = 0.28$, paired sample t-test) (D) Proportion of *across*-stimulus saccades (*within*-stimulus saccades excluded) that land on each of the six image categories (0.27 vs. 0.26 for human vs. monkey respectively; $p = 0.39$, paired sample t-test). (A-D) Error bars indicate the 95% confidence interval of the mean.

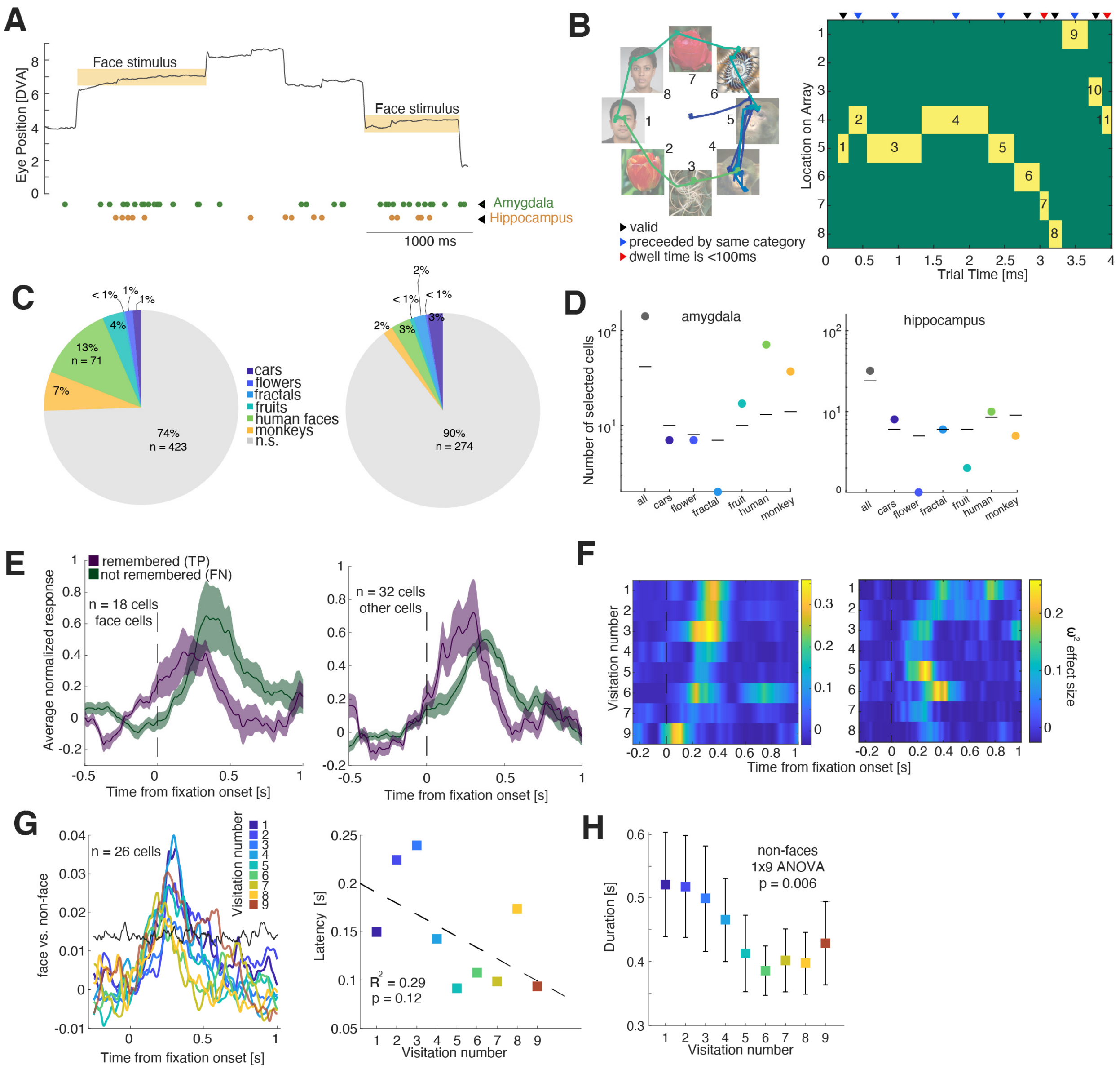

Figure S2. Effect of image category and stimulus familiarity on the latency of visually selective responses in the amygdala.

(A) Example eye trace (x-coordinate shown only) and two concurrently recorded cells, one in amygdala and one in hippocampus, that are selective for images of human faces. (B) Selection criteria for fixations, demonstrated through an example trial. For each fixation, we label its spatial location on the array (1-8), and its temporal location in the sequence (1-11). Note that only the fixations marked as "valid" are used in the single-unit analysis shown in the remaining panels and in Figure 2. (C) Proportion of visually selective cells for amygdala (left) and hippocampus (right). (D) Significance testing for the proportion of visually selective cells. We compare the observed proportion with the 99thpercentile of the null distribution (indicated with the solid horizontal line). The null distribution was created by shuffling the image category of fixated stimuli, and repeated the selection procedure 1000 times. Note that the significance threshold for each category is different because not all categories were present in each session. (E) Average activity of 18 face cells, measured during the learning phase, for faces that were

remembered and not remembered during the testing phase. The difference between this analysis and that show in Figure 2E-F, is that here we only include cells that contribute to both the remembered and not-remembered face groups. (F) Image category effect sizes (face vs. non-face) for fixation groups of varying ages (see Methods). The cell on the left is the same as that shown in Figure 2H. Note that while we used 9-bins to group fixations by their corresponding age (see Methods), some of these bins might be empty for some of the face selective cells, due to our fixation-selection procedure. (G) Similar to Figure 2K, but with cells selective for cars and monkey images (the two image categories for which memory performance was the worst, see Figure 2C) removed. (H) Despite the lack of neural evidence of the effect of familiarity on coding latency, as seen in (G), subject still dwell significantly longer on novel images.

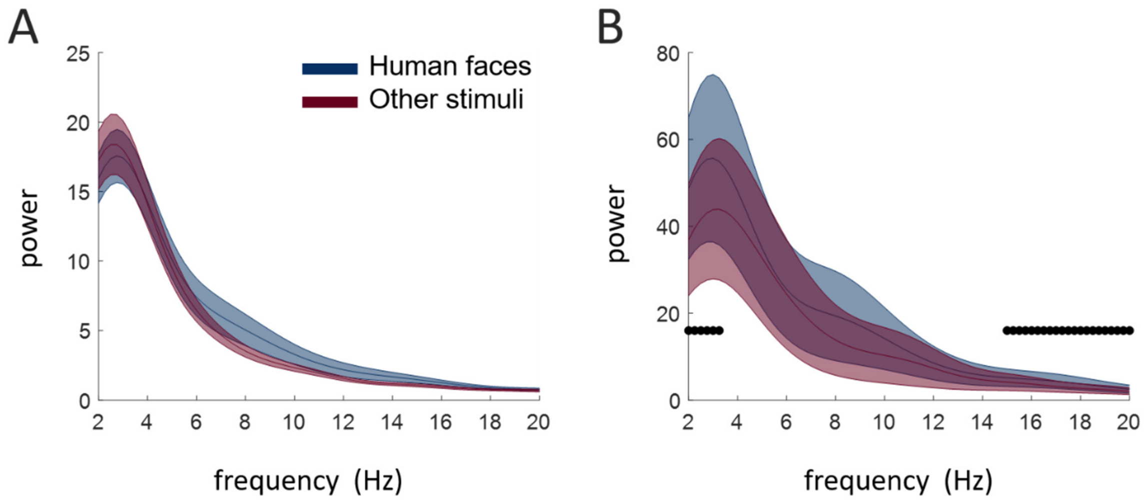

Figure S3. Category-dependent modulation of power in Amygdala and Hippocampus. (A) Power in Amygdala is not different when fixating human faces versus other stimuli. (B) Power in the Hippocampus shows differences when fixating human faces versus other stimuli, but not in the frequency range around 6.5 Hz. Shading depicts SEM. Black dots indicate contiguous frequency bins sowing significant differences.

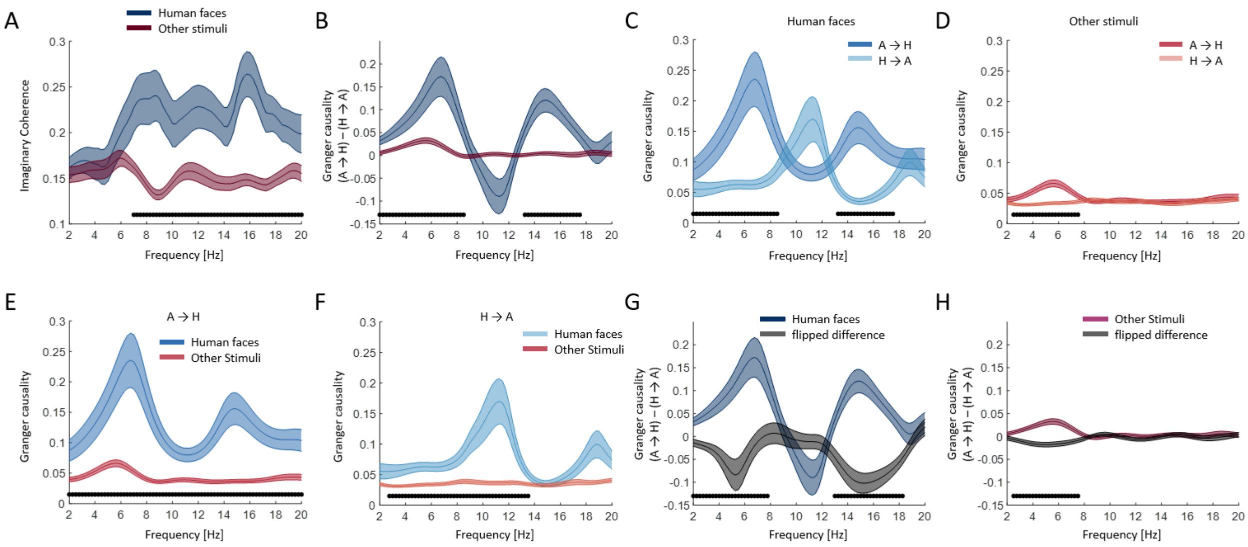

Figure S4. Controlling for power differences between regions.
To investigate whether our main effects (Amygdala drives Hippocampus at 6.5 Hz) could be driven by signal-to-noise differences between regions, we excluded 10% of trials with the largest power difference (Amygdala minus Hippocampus). We found patterns of Amygdala-Hippocampus interactions highly similar to our main results. (A) Increased amygdala-hippocampus imaginary coherence for human faces versus other stimuli. (B) Granger causality differences (A -> H minus H -> A) are significantly larger when fixating human faces compared to fixating other stimuli. Differences peak at 6.5 Hz and 15 Hz. (C) Granger causality spectra when fixating human faces, indicating amygdala drives hippocampus (peaks at 6.5 Hz and 15 Hz). (D) Granger causality spectra when fixating other stimuli, indicating amygdala drives hippocampus, but to a lesser extent (peak at 5.5 Hz). (E) Granger causality spectra (A -> H) for fixation on human faces compared to fixations on other stimuli, indicating a larger Granger causal influence of amygdala on hippocampus during fixations on faces with peaks at 6.5 Hz and 15 Hz. (F) Granger causality spectra (H -> A) for fixation on human faces versus fixations on other stimuli, indicating a larger Granger causal influence of hippocampus on amygdala during fixations on faces peaking at 11 Hz. (E&F). Note that these two panels re-plot the same data shown in C&D for ease of comparison. (G & H) When flipping the time series, the directions of the Granger causality interactions were reversed. (G) Difference (A -> H minus H -> A) in Granger causality for fixations on human faces. (H) Difference (A -> H minus H -> A) in Granger causality for fixations on other stimuli. Shading depicts SEM. Black dots indicate contiguous frequency bins showing significant differences (corrected for multiple comparisons across frequencies). Post-hoc t-tests confirmed that power was not significantly different between conditions in at the frequency of interest (6.5 Hz), $p > 0.27$ in Amygdala and Hippocampus, respectively.

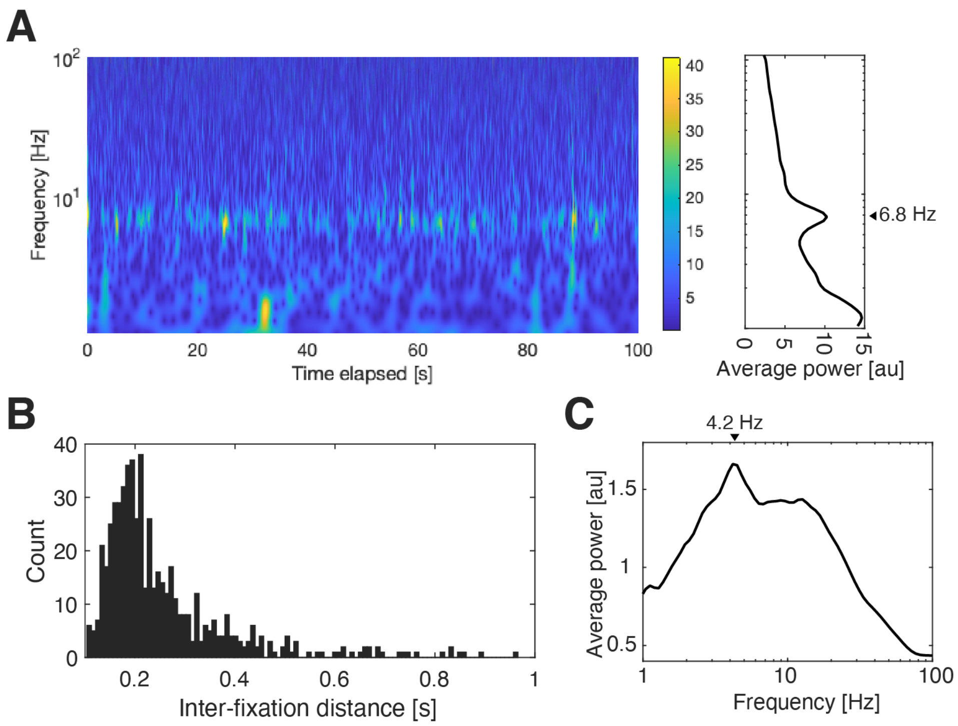

Figure S5. Spectrogram from example session

(A) Example spectrogram from a hippocampal electrode in a 100-second snippet recorded during an example session. Power in difference frequency bands was computed using the continuous wavelet transform, with frequencies sampled logarithmically from 1-250 Hz (cut off at 100 Hz here for clarity). The panel on the right shows the average power in different bands, with a peak at 6.8 Hz. (B) For the same session, we show the distribution of inter-fixation distance, as a measure of the rhythmicity with which the subject was sampling images (~4 fixations/second). (C) Power spectral density of the velocity profile of the subject's eye-movements. The peak of the spectra is at 4.2 Hz, and therefore the rhythmicity of the eye movement cannot explain the peak in the LFP spectra shown in (A).

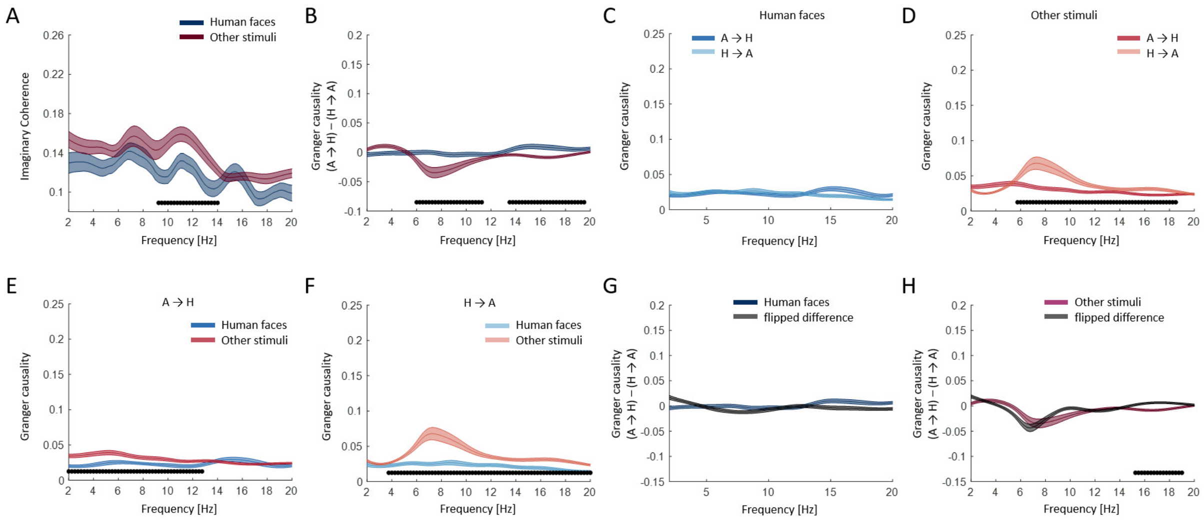

Figure S6. Contralateral connectivity.
We computed imaginary coherence (iCoh)and Granger causality (GC) for contralateral Amygdala-Hippocampus pairs (i.e., left Amygdala and right Hippocampus; right Amygdala and left Hippocampus). The results differ substantially from our main results, which were computed unilaterally (within a hemisphere). The GC difference between contralateral pairs for human faces is only spurious, since a time-reversal did not result in a reversal of GC (G). Similarly, GC difference between contralateral pairs for other stimuli is only non-spurious for frequencies between ~15 and 19 Hz. No significant difference for human faces is found when comparing directionalities (C). When comparing human faces to other stimuli, the Amygdala does not drive the Hippocampus more for faces. Overall, GC values are substantially smaller than and do not reflect the general pattern of the main results. iCoh patterns are flipped (Human faces < other stimuli), compared to the main results. Note that y-limits in all panels were adjusted to match Figure 3 (main text).

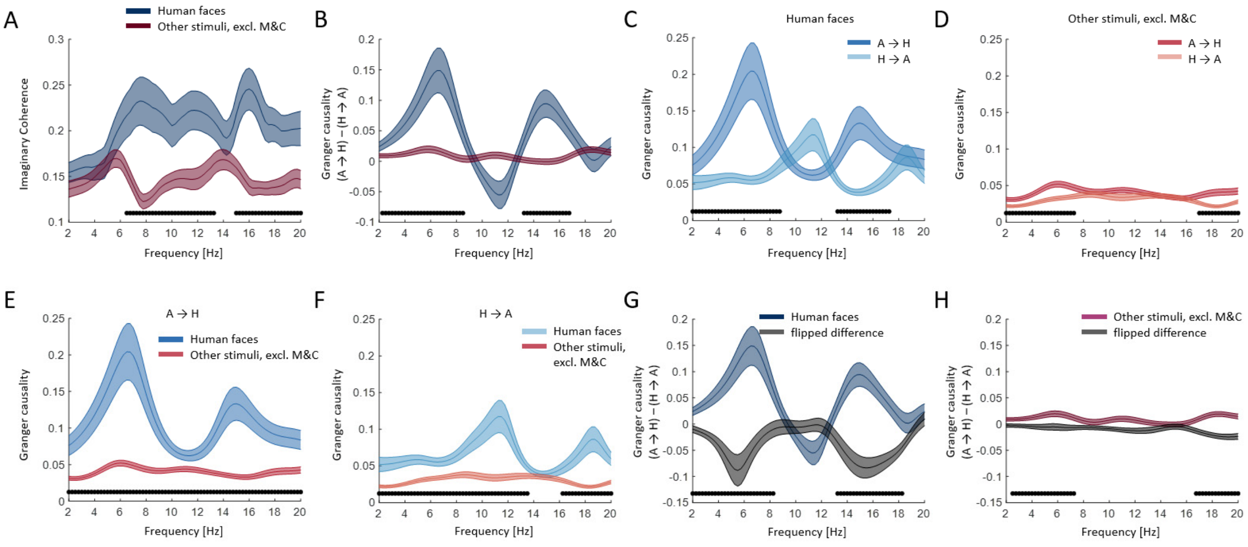

Figure S7. Human faces versus other stimuli excluding monkey faces and cars. Behavioral results showed that some subcategories (monkey faces and cars) are remembered less than faces (see Fig. 2C). Here, we excluded these subcategories from the 'other stimuli' and compared their connectivity profiles to connectivity for human faces, to investigate whether our main analyses are influenced by memory performance for subcategories. We found patterns of Amygdala-Hippocampus interactions highly similar to our main results. (A) Increased amygdala-hippocampus imaginary coherence for human faces versus other stimuli. (B) Granger causality differences (A -> H minus H -> A) are significantly larger when fixating human faces compared to fixating other stimuli. Differences peak at 6.5 Hz and 15 Hz. (C) Granger causality spectra when fixating human faces, indicating amygdala drives hippocampus (peaks at 6.5 Hz and 15 Hz). (D) Granger causality spectra when fixating other stimuli, indicating amygdala drives hippocampus, but to a lesser extent (peak at 5.5 Hz). (E) Granger causality spectra (A -> H) for fixation on human faces compared to fixations on other stimuli, indicating a larger Granger causal influence of amygdala on hippocampus during fixations on faces with peaks at 6.5 Hz and 15 Hz. (F) Granger causality spectra (H -> A) for fixation on human faces versus fixations on other stimuli, indicating a larger Granger causal influence of hippocampus on amygdala during fixations on faces peaking at 11 Hz. (E&F). Note that these two panels re-plot the same data shown in C&D for ease of comparison. (G & H) When flipping the time series, the directions of the Granger causality interactions were reversed. (G) Difference (A -> H minus H -> A) in Granger causality for fixations on human faces. (H) Difference (A -> H minus H -> A) in Granger causality for fixations on other stimuli. Shading depicts SEM. Black dots indicate contiguous frequency bins showing significant differences (corrected for multiple comparisons across frequencies).

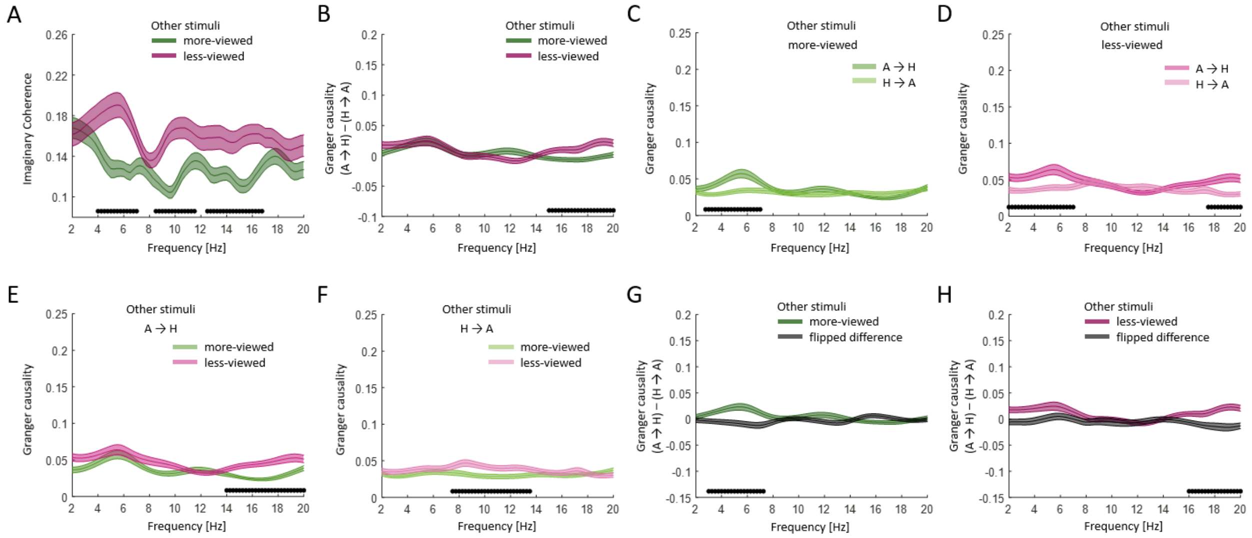

Figure S8. 'More-viewed' vs. 'less viewed' non-face stimuli.
We computed imaginary coherence (iCoh)and Granger causality (GC) for 'more-viewed' vs. 'less viewed' non-face stimuli, by splitting the non-face trials based on the total number of fixations, while keeping our criteria for saccades and number of trials.
(A) Comparing 'more-view' vs. 'less viewed' showed a significant difference for iCoh, however, the direction of this difference is inverted compared to our main results (see Fig. 3B): 'less viewed' trials show higher imaginary coherence than 'more-viewed' trials, which speaks against the notion that this condition difference is driving our main results. (B,E) The comparison of 'more-viewed' vs. 'less viewed' non-face stimuli showed no differences in the frequencies of interest (6.5 Hz), neither for GC difference (B) nor for the critical GC direction (Amygdala drives Hippocampus; E). Note that y-limits in all panels were adjusted to match Figure 3 (main text).

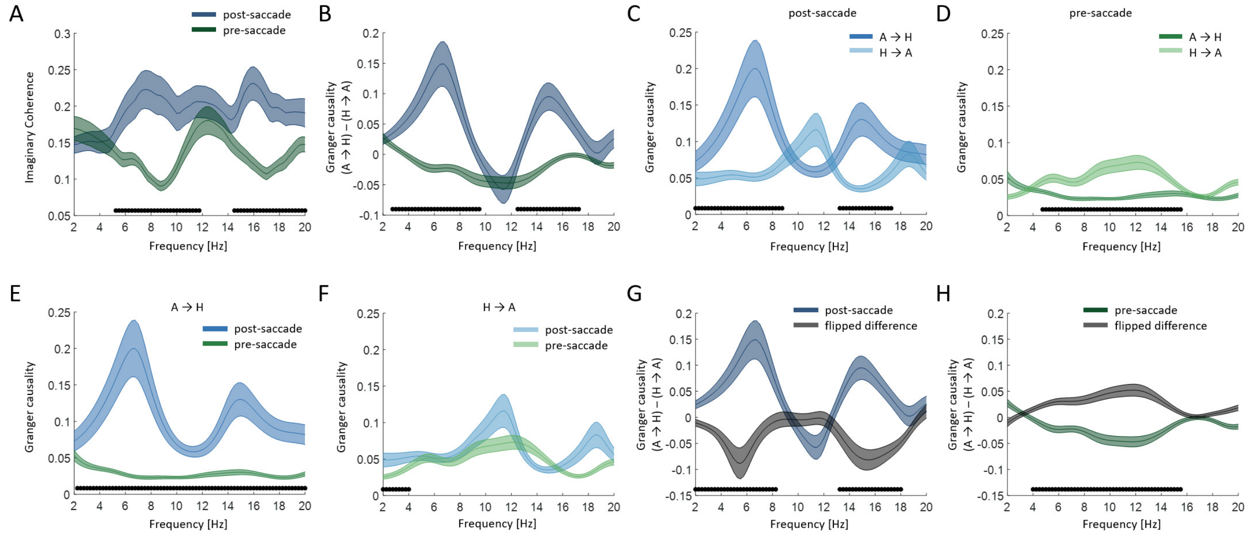

Figure S9. 'Post-saccade' vs. 'pre-saccade' connectivity.

We computed imaginary coherence (iCoh)and Granger causality (GC) prior to saccade onset and compared them to our main results, where we computed iCoh and GC post-saccade. Post-saccade iCoh and GC was found to be significantly larger than pre-saccade iCoh and GC.

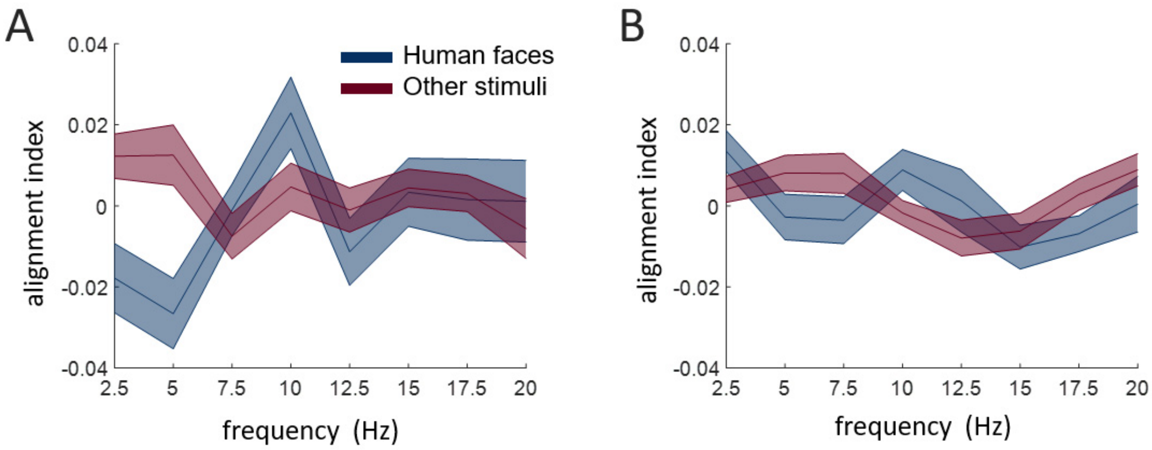

Figure S10. Fixation onset related phase alignment.
(A) Hippocampal fixation-related phase alignment is not significantly different for fixations on human faces versus fixations on other stimuli. (B) Amygdala fixation- related phase alignment shows no significant difference between conditions. Shading depicts SEM. Analyses and statistics are the identical to those used for Fig. 4A&B, except that here fixation onsets were used to align the signals, instead of saccade onsets in Fig. 4A&B.
